# Endometrial Immune Dysfunction in Recurrent Pregnancy Loss

**DOI:** 10.3390/ijms20215332

**Published:** 2019-10-26

**Authors:** Carlo Ticconi, Adalgisa Pietropolli, Nicoletta Di Simone, Emilio Piccione, Asgerally Fazleabas

**Affiliations:** 1 Department of Surgical Sciences, Section of Gynecology and Obstetrics, University Tor Vergata, Via Montpellier, 1, 00133 Rome, Italy; pietropolli@med.uniroma2.it (A.P.); piccione@med.uniroma2.it (E.P.); 2 U.O.C. di Ostetricia e Patologia Ostetrica, Dipartimento di Scienze della Salute della Donna, del Bambino e di Sanità Pubblica, Fondazione Policlinico Universitario A.Gemelli IRCCS, Laego A. Gemelli, 8, 00168, Rome Italy; nicoletta.disimone@policlinicogemelli.it; 3 Istituto di Clinica Ostetrica e Ginecologica, Università Cattolica del Sacro Cuore, Largo A. Gemelli 8, 00168 Rome, Italy; 4 Department of Obstetrics, Gynecology, and Reproductive Biology, College of Human Medicine, Michigan State University, Grand Rapids, MI 49503, USA; FAZLEABA@msu.edu

**Keywords:** recurrent pregnancy loss, endometrium, decidua, immunologic dysfunction

## Abstract

Recurrent pregnancy loss (RPL) represents an unresolved problem for contemporary gynecology and obstetrics. In fact, it is not only a relevant complication of pregnancy, but is also a significant reproductive disorder affecting around 5% of couples desiring a child. The current knowledge on RPL is largely incomplete, since nearly 50% of RPL cases are still classified as unexplained. Emerging evidence indicates that the endometrium is a key tissue involved in the correct immunologic dialogue between the mother and the conceptus, which is a condition essential for the proper establishment and maintenance of a successful pregnancy. The immunologic events occurring at the maternal–fetal interface within the endometrium in early pregnancy are extremely complex and involve a large array of immune cells and molecules with immunoregulatory properties. A growing body of experimental studies suggests that endometrial immune dysregulation could be responsible for several, if not many, cases of RPL of unknown origin. The present article reviews the major immunologic pathways, cells, and molecular determinants involved in the endometrial dysfunction observed with specific application to RPL.

## 1. Introduction

Recurrent pregnancy loss (RPL) is the loss of two or more pregnancies before 24 weeks of gestation, according to the European Society of Human Reproduction and Embryology [1]. The Practice Committee of the American Society for Reproductive Medicine defines RPL as two or more failed clinical pregnancies [2]. RPL is a challenge for the clinical and scientific community. In fact, in only around 50% of RPL cases can defined causes/risk factors can be found, including advanced maternal age, genetic abnormalities, selected maternal autoantibodies, endocrine dysfunctions, and uterine abnormalities [3]. The remaining RPL cases currently are unexplained (uRPL) [3,4]. 

It is believed that a significant, although not exactly quantified, proportion of RPL is associated with immune etiologies [5] and that in these cases the pregnancy losses can occur through persistent disturbances in several immune pathways [6]. In this context, a relevant role could be played by the endometrium.

The endometrium has a crucial role in reproduction. It is the maternal tissue that comes into direct contact with the embryo and allows for its proper implantation, survival, and development, processes in which it actively participates. Extensive investigation has been carried out in the last decades to clarify the biomolecular mechanisms which make the endometrium receptive to the embryo, as well as the specific cell types and the cellular pathways involved in endometrial receptivity. However, our knowledge on these mechanisms is still largely incomplete. The currently available information indicates that the endometrium is a unique tissue in which a series of events—collectively called decidualization—occur in order to ensure, in a dynamic fashion, the correct environment for the developing conceptus. Emerging evidence indicates that a pivotal role in endometrial remodeling and maternal tolerance towards the embryo is played by several cells of the innate and adaptive immune system, which have or acquire specific characteristics when they are in or reach the endometrium, together with a growing number of immunoregulatory molecules. 

The experimental and clinical evidence also suggests that derangements occurring in the endometrial immune environment can be involved in several important reproductive dysfunctions, such as recurrent implantation failure (RIF) and recurrent pregnancy loss (RPL) of otherwise unexplained etiology. This review summarizes the major contributions of the immune system in the physiology of the endometrial function and the current knowledge on the major known alterations of the endometrial immune system with specific application to RPL in women.

## 2. Physiological Endometrial Function and the Immune System

### 2.1. Endometrial Remodeling and Decidualization 

The endometrium is the maternal tissue that comes into direct contact with the embryo, which is immunologically different from the mother, being usually considered as a semi allograft, or even as a complete allograft in the case of oocyte donation [7]. The major function of the endometrium is to create and ensure an optimal endocrine/paracrine, immune, and molecular environment that allows for proper apposition, attachment, implantation, invasion and development, and full maturation of the embryo. To accomplish this mission, the endometrium needs to develop a series of unique and striking adaptive changes collectively termed decidualization, resulting in profound morphological and functional reprogramming of the endometrial stromal cells that differentiate into highly specialized cells with secretory capabilities. Among the eutherian—placental mammals—these changes are particularly significant in species with an invasive type of placenta, specifically in humans who have the most invasive type of placentation. In fact, the extent of decidualization is proportional to the degree of invasiveness of the embryo [8]. While endometrial transformation in most animal species occurs in response to the presence of the embryo, in a few species—including humans—it takes place well in advance of the presence of the embryo and, therefore, is—at least initially—under exclusive maternal hormonal control. If conception does not occur, in response to falling circulating levels of progesterone the decidualized endometrium in these species is shed and menstruation ensues. This initiates the beginning of a new cycle. 

The majority of recent information on the role of the endometrium and on the functional changes occurring in the normal endometrium stems from the histologic investigations performed on human endometrial biopsies, as well as from studies carried out in vivo in animal species (particularly baboons and gene knockout mice) and in vitro in primary cell cultures of human endometrial stromal cells (HESCs). 

Despite the considerable differences among the species, the overall emerging picture is that decidualization of the endometrium is a process involving profound cell reprogramming, tissue remodeling, changes in gene expression and post-translation regulation, and alterations in cell signaling pathways. Considerable changes and modulation of the activity of the immune cells at a local level also occurs. These events involve a large number of molecular mediators and effectors. A summary of the major endometrial changes characteristic of decidualization in women is described in Table 1. 

The detailed description of all the changes and the mediators involved in decidualization is beyond the scope of the present article and has been discussed in several excellent reviews recently [9,10,11,12,13]. Here, the major focus is to evaluate the significance of the endometrial changes, particularly decidualization, in relation to the role played by the immune system. 

Decidualization can be evolutionarily considered to have two, not mutually exclusive functions. On the one hand, it can be considered as the biological solution of a maternal–fetal conflict in which the invasiveness of the embryo, aimed to maximally ensure the expansion of fetal genes (partially different from the maternal ones) is contrasted by the need of maternal genes to ensure their own expansion in the present and future pregnancies [14]. On the other hand, it can be viewed as the adaptive response to the maternal need of controlling its relevant investment in a pregnancy bearing a single, high quality fetus for a prolonged period of time [15]. This concept is supported by robust evidence indicating that the decidualized endometrium acts as a biosensor of the quality of the embryo [16,17], that implantation is characterized by a cooperation between the embryo and the decidualized endometrium [18], and that the loss of endometrial plasticity can be implicated in unexplained RPL [19]. Many key events occurring in the endometrium during decidualization implantation and in the decidua throughout pregnancy are realized with the relevant contribution of the immune cells and are mediated by many immunoregulatory molecules. Emerging evidence suggests that derangements in the normal immune function can occur in these tissues in subsets of women with RPL.

### 2.2. Immune Cells and Their Functions in the Normal Endometrium and Decidua

**Figure 1:**
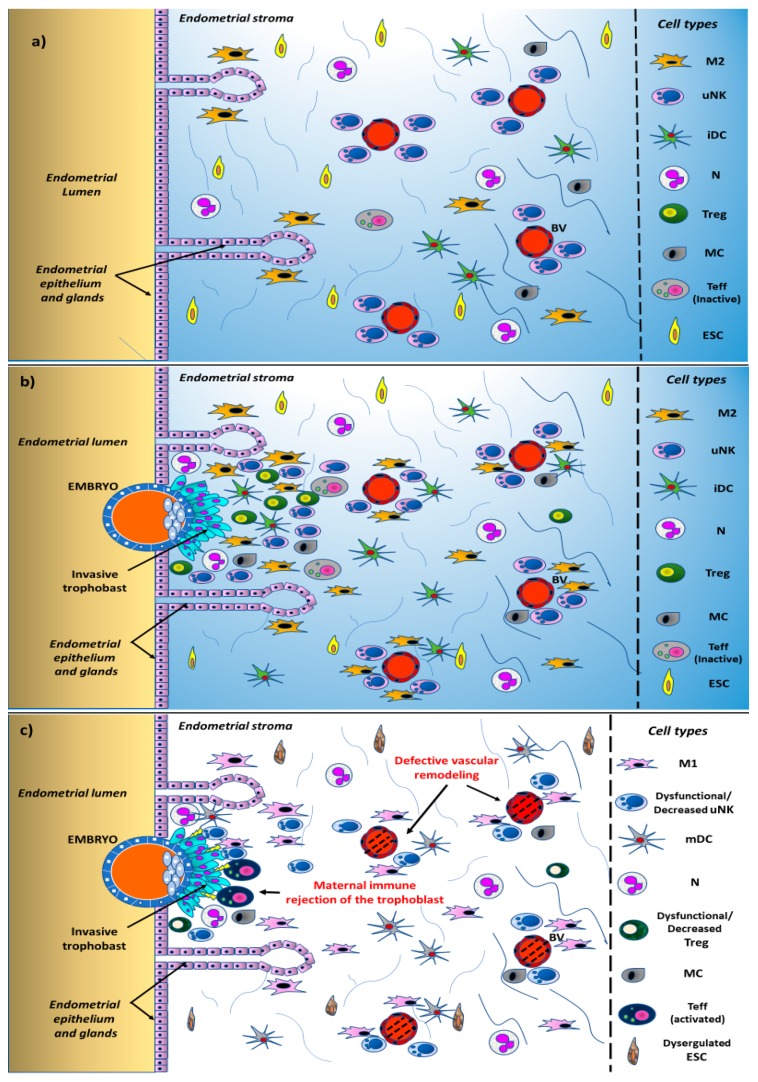
Schematic representation of the changes occurring in the human endometrium and in local immune cell trafficking in the normal state and in recurrent pregnancy loss (RPL). (**a**) Endometrium in the secretory phase of the menstrual cycle in the absence of the embryo; (**b**) endometrium in the presence of a normally implanting embryo; (**c**) endometrial immune derangements in RPL. M: macrophages; uNK: uterine natural killer cell; iDC: immature uterine dendritic cell; mDC: mature uterine dendritic cell; N: neutrophil granulocyte; Treg: regulatory T cell; MC: mastocyte; Teff: effector T cells; ESC: endometrial stromal cell; BV: blood vessel.

A great body of experimental work has been carried out to investigate the specific populations of immune cells in the endometrium throughout the menstrual cycle, during implantation, and early pregnancy; their specific roles in early pregnancy together with the regulatory molecules that are expressed are being characterized. This information is summarized in Table 2 and Table 3. Likewise, a growing number of mechanisms are proposed to explain the development of maternal tolerance towards the immunologically different conceptus. These proposed mechanisms—schematically summarized in Table 4 and illustrated in Figure 1a,b—could be the basis to explain, understand, and, possibly, treat the immune-mediated RPL, that likely represent a substantial proportion of all unexplained RPL. However, it is actually extremely difficult to disentangle the most important mechanisms from the accessory ones. The emerging overall picture strongly suggests that in normal pregnancy, the maternal immune system undergoes considerable modulation in at least many (if not all) key components in order to develop tolerance towards the foreign paternal antigens of the fetus and an immunologically favorable environment for the fetus while at the same time maintaining full responsive capacity against other foreign antigens [20,21,22,23]. The pregnancy-related changes in the immune cells and system occurring in the endometrium and decidua are only partially determined. Nevertheless, with regard to the success of implantation, the most important changes known so far in the endometrial immune cells involve macrophages, uterine natural killer (NK) cells, dendritic cells (DCs), T cells, especially cytolytic *T cells* (CTLs), whose specific roles have been, at least in part, determined.

#### 2.2.1. Macrophages

Macrophages occur in low numbers in the non-pregnant endometrium, even though their numbers substantially increase in the luteal phase of the cycle. Their number dramatically increases as pregnancy occurs, reaching 20%–25% of all leukocytes in the decidua, as shown in Table 2. There is evidence that the macrophages in the decidua differentiate toward an immunoregulatory M2 type, since they express several M2 markers [81]. The possibility has been raised that also a proinflammatory subset of macrophages is present, although to a much lesser extent, in the early decidua [82]. During early pregnancy, macrophages are localized mainly close to invading trophoblast cells and to the spiral arteries [83] and are believed to play several relevant roles and functions in early pregnancy: enhancement of blastocyst implantation and trophoblast invasion, remodeling of spiral arteries, clearance of apoptotic cells and cell debris, as well as protection of the fetus against foreign pathogens [30,31,32,33,34,35,81,84,85]. It has been shown that decidual spiral artery remodeling begins before cellular interaction with cytotrophoblasts [86] and that matrix remodeling in spiral arteries is initiated by infiltrating leucocytes, including macrophages, while extravillous trophoblasts (EVTs) become involved at later stages [87]. Four discrete stages of vascular remodeling have been described according to the extent of smooth muscle cells disruption in human decidual vessels [88]. Vessel infiltration by macrophages has been found to be more prominent in the intermediate stages II and III [88]. 

#### 2.2.2. NK Cells

NK cells are lymphocytes with multiple biologic actions including cytotoxicity and cytokine-producing capacity [89]. In the peripheral blood, two populations of NK cells are present—the CD56 ^dim^ CD16^+^ NK, that are cytotoxic toward tumors and virally infected cells and comprise 90% of circulating NK cells, and the CD56^bright^CD16^−^ NK, whose main activity is regulatory through the secretion of cytokines [81,89]. Decidual or uterine NK (uNK) cells which are present in the decidua are distinct from peripheral blood NK (pbNK) cells [83,90]. They constitute 50%–90% of lymphocytes in human uterine decidua in early pregnancy. A comparative microarray analysis demonstrated that the uNK cells are distinctly different from pbNK cells and have an increased expression of a number of surface proteins, including lectin receptors, killer cell Ig-like receptors, tetraspanins, and integrin subunits. In addition, the secreted immunomodulatory molecules, galectin-1 and glycodelin (PAEP), were also selectively expressed on the dNK cells [91]. Uterine NK (uNK) are the predominant leukocyte population in the endometrium, increase progressively throughout the menstrual cycle, and are maximal in early pregnancy, being ~75% of the decidual leukocyte population [27]. uNK are phenotypically similar, although not identical, to circulating CD56^bright^CD16^−^ [91,92,93] and during early pregnancy are found adjacent to the invading trophoblast cells [88,94]. This observation, together with the finding that activated uNK can produce angiogenic factors (VEGF and ANG2) and a vast array of cytokines, including GM-CSF, CSF-1, TNFα, IFN-γ, TGF-β, LIF, IL2, CXCL10, and CXL12 [91], suggest a role of uNK in promoting trophoblast invasion, protecting the embryo from maternal immune attack, and enhancing angiogenesis [88,95]. The role of decidual natural killer cells (dNK) in regulating trophoblast invasion and the maternal immune response against trophoblasts is also strongly suggested by the observation that dNK, or uNK cells, in addition to their specific location in close proximity to the trophoblast, also express the specific appropriate receptors for those human leukocyte antigens (HLAs) that are uniquely expressed by the trophoblasts, i.e., the killer immunoglobulin receptor (KIR) for HLA-C, the CD94/NKG2A for HLA-E, and the ILT2 for HLA-G [70,96]. The combined interaction between the dNK/uNK cells and the HLAs of trophoblasts is believed to be a potential key factor involved in the prevention of the maternal immune rejection of the conceptus [72,73,97,98]. 

#### 2.2.3. DCs

Dendritic cells have a dual role—they can differentiate into potent antigen presenting cells which can activate effector T cells or, in their immature state, they can enhance immune tolerance by inducing the generation of Tregs [99]. In the decidua, DCs are believed to play an important role in maternal recognition of paternal antigens in both pre- and implantation periods. In particular, the seminal fluid has the ability to recruit DCs (as well as macrophages) to the decidua; DCs, in turn, can take up and present the seminal soluble major histocompatibility complex (MHC) T cells in the regional draining lymph nodes and induce the expansion of Tregs population [21,100,101]. DCs in a specific cytokine environment, characterized by the predominance of G-CSF, GM-CSF, IL-4, IL-10, and TGFβ and in the presence of IDO, acquire the phenotype of tolerogenic DCs (tDCs) and become able to drive the differentiation of naïve Th0 cells towards tolerogenic regulatory T cells (Tregs) rather than towards cytotoxic effector T cells [33,54]. In addition to the above immunological actions, DCs have been suggested to play a relevant trophic role by enhancing both endometrial stromal cell differentiation and proliferation and local angiogenesis [102]. 

#### 2.2.4. Tregs

Tregs (CD4+CD25+Foxp3+) are a subset of CD4+ T cells and are important components of adaptive immunity since their major function is to limit immune reaction. Tregs are involved in immunological self- and transplant tolerance and play a relevant role in preventing autoimmune responses against self antigens [103,104]. Therefore, the possibility that these functions of Tregs might be instrumental in the proper immunologic relationship between the mother and the embryo has been raised, and recent convincing evidence defines the potential key role of these cells in early pregnancy [21,33,54,65,105]. It has been shown, in both mice and in humans, during normal pregnancy there is an expansion in the population of decidual Tregs, which exert several pregnancy-promoting actions [54]: prevention of the immune rejection of the fetal paternal antigens by effector T cells, induction of decidual support for embryo implantation through their action on other leukocyte and non-leukocytes cell types [106], and by enhancing proper maternal vascular remodeling [107]. There is evidence supporting the concept that the pregnancy-promoting actions of Tregs are particularly relevant in the peri-implantation period and in the very early stages of pregnancy, whereas the role of Tregs in later stages appears to be more limited [108]. 

#### 2.2.5. Other Components of the Immune System

The role of other components of the immune system, such as granulocytes, CD8+ cells, and B cells, has been scarcely explored to date, particularly in relation to RPL; further investigation is needed to better determine the potential relevance of these important components of innate and adaptive immunity. However, with specific application to B cells and plasma cells, emerging data support the concept that regulatory B (Breg) cells—a subset of B cells with immunosuppressive properties mainly studied in autoimmunity, cancer, and transplantation tolerance—can play a role also in pregnancy since they are a major cellular source of the powerful anti-inflammatory cytokine IL-10. Moreover, Breg cells induce suppression of other immune cell populations and can enhance induction and maintenance of Tregs [109]. A reduction in the number of Breg cells has been observed in women with spontaneous abortion [110]. In normal pregnancy, hCG induces the expansion of Bregs, as well as their IL-10 production; moreover, hCG enhances the generation of plasma cells capable to produce specific pregnancy-protective antibodies [111]. 

### 2.3. Cytokine/Chemokine Network and the Maternal–Fetal Immune Cross-Talk

The molecular dialogue between the trophoblast, the decidua, and the maternal immune cells aimed at the proper control of embryo implantation and early pregnancy progression is mediated by a network of cytokines and chemokines. The extensive research carried out so far in this field has revealed that a vast and continuously increasing array of cytokines and chemokines could be potentially involved, to various degrees, in the immune and non-immune relationship between the mother and the conceptus. The cytokines suggested to play a role in this context include at least IL-1β, IL-2, IL-4, IL-5, IL-6, IL-8, IL-9, IL-10, IL-11, IL-12, IL-13, IL-15, IL-17, IL-18, IL-22, IL-23, IL-34 LIF, TGFβ, G-CSF, GM-CSF, TNF-α, and IFN-γ [21,23,33,99,112,113,114,115,116,117]. The chemokines potentially involved include at least CCL2, CCL3, CCL4, CCL5, CCL17, CCL19, CCL20, CCL22, CXCL8, CXCL9, CX3CL1, CXCL10, CXCL12, and CXCL16 [40,99,118,119]. The high number of cytokines and chemokines believed to participate at least to some extent in maternal–fetal interactions, the pleiotropic actions of many of the above molecules and their redundant and often overlapping effects make it extremely difficult to clearly disentangle the actual role of each molecule. Despite these limitations, the function of selected cytokines and chemokines in the immunologic interaction between the mother and the trophoblast is being discovered in several cases. For instance, the importance of LIF in early stages of embryo implantation has become clear [120]; moreover, emerging evidence strongly suggests that on the one hand, the major pro-inflammatory cytokines IL-1β and TNF-α, released by uterine macrophages sensitized by seminal fluid antigens, contribute to the development of tolerogenic DCs (tDCs) [54]; on the other hand, after embryo implantation, the anti-inflammatory cytokines IL-10 and TGFβ play key roles in the regulation of tolerogenic DCs in the decidua, in reducing the local inflammatory response and in driving the expansion of Treg cells [33,54]. Among the chemokines, CXCL1 was found to be markedly upregulated in decidualized human endometrial stromal cells [121]. The large body of experimental work carried out so far supports the general concept that the cytokines, the chemokines, and their receptors can be expressed and produced by the glandular epithelial and stromal cells of the endometrium, by the decidual cells, by the cellular components of the innate and acquired immunity, as well as by the trophoblast cells; their network creates a local microenvironment of paramount importance for the successful implantation of the embryo, for the regulation of trophoblast migration, and, therefore, for the proper development of the pregnancy. Further investigation is still needed: (1) to clearly define the specific contribution of each of the above molecules in the maternal–fetal immune dialogue; (2) to verify the applicability of the information obtained in animal models to human pregnancy, taking into account the differences between the species [99].

## 3. Immune Dysregulation in the Endometrium and Decidua in RPL

The evidence indicating the fundamental role of proper immunologic dialogue between the mother and conceptus in the normal implantation and pregnancy development has prompted a large number of studies aimed at investigating the impact of dysregulations in the maternal–fetal immune relationship in RPL. This is particularly so for the so-called “unexplained” RPL (uRPL), which accounts for around 50% of the cases of RPL. Many of the abnormalities found in this context occur within the endometrium and the decidua, which is the major maternal–fetal interface. In this section, for reasons of clarity, the relevant findings will be divided according to the specific cell types involved and then to the overall network of immunoregulatory molecules.

### 3.1. Endometrial Cells in RPL with Specific Application to the Regulation of the Local Immune Function

It has been suggested that there are evolutionary adapted checkpoints during early pregnancy which are designed to reject a pregnancy if the conceptus or the receptive endometrium is compromised [13]. An inadequately developed decidualized endometrium is one of them. Several studies have demonstrated that the transformation of the stromal fibroblasts into the decidual phenotype is compromised in RPL patients [19,122,123]. The importance of a proper decidualization of endometrial stromal cells in implantation and early pregnancy maintenance has been elucidated by a series of observations supporting the concept that endometrium is a fine biosensor of the quality of the implanting embryo [15,17,19]. Specifically, studies carried out on human endometrial stromal cells have shown that these cells, when differentiating into decidual cells, become sensitive to embryonic signals to whom they respond differently according to the quality of the embryo. In normal women, low-quality embryos inhibit the secretion of factors playing a key role in implantation; conversely, developmentally competent embryos produce signals promoting implantation [16]. These studies provide experimental evidence that the endometrium of normally fertile women is selective towards the embryo and that low-quality embryos undergo an early rejection. Conversely, the endometrium of women with RPL is less sensitive and allows the implantation of low-quality embryos, which will be rejected later in pregnancy. This hypothesis is further supported by the clinical observation that women with RPL have a shorter time-to-pregnancy—the time needed to become pregnant—than normally fertile women [122,124]. Even though the specific mechanisms underlying these derangements are still uncertain, it is likely that the disordered decidual response observed in women with RPL involves immune system mediators and cells, since it is characterized by the failure of endometrial stromal cells to transit from an initially proinflammatory phenotype to an anti-inflammatory one during decidualization, as normally occurs in physiological conditions [9]. 

### 3.2. Immune Cells in the Endometrium in RPL

#### 3.2.1. Macrophages

There is scant information on the role of macrophages in RPL. Very recent experimental evidence shows that in women with uRPL, no reduction of M1 decidual macrophages can be detected [31], in contrast to what occurs in normal pregnancy. This finding suggests that in women with uRPL, there would be a limited differentiation of macrophages toward the M2 immunoregulatory polarization. This is in agreement with the results of a previous study, in which decidual macrophages from women with uRPL showed an increased expression of CD80, CD86, and a lower expression of IL-10 compared with control women [125]. These findings suggest that the macrophage regulation capacity of Tregs, mediated by TGFβ and by cell–cell contact, is decreased in women with uRPL [125]. However, further investigation is needed to further clarify the role of macrophages in RPL [85], since similar reduction in the M2 macrophage population has been also observed in women with spontaneous sporadic miscarriage [31].

#### 3.2.2. uNKs

Considerable interest in the role of uNKs in pregnancy complications, and particularly in RPL, reflects the recognized importance of these cells in implantation and in early pregnancy. Overall results indicate that RPL is associated with several/different abnormalities in uterine/decidual NK cell number and function, although some controversy still exists. Most of the studies support the concept that in uRPL, the pregnancy-promoting functions of uNK are dysregulated in several ways. Indeed, in women with uRPL, higher concentrations of uNK have been detected than in normal fertile controls [92,126,127,128,129,130,131], even though this finding has not always been consistent [132]. In other studies, despite no differences in the proportion of endometrial NK cells between controls and women with RPL, women with RPL have a significant decrease in the CD16^−^CD56^bright^ NK cell subset, that is normally found in uncomplicated pregnancies [133]; this suggests that uNK cells in women with uRPL are quantitatively and qualitatively different from those normally found. Accumulating evidence supports this hypothesis. For instance, it has been shown that women with uRPL have increased endometrial populations of cytotoxic CD16^+^uNK [92] and of uNK expressing the natural cytotoxicity receptors NKp46, NKp44, and NKp30 at higher levels than those detected in control women and in women with unexplained infertility or recurrent implantation failure [28,92,134,135]. This suggests that the cytotoxic activity of uNK is higher in RPL than in control women. Even though the results are not always unequivocal, it is becoming clear that uNK has different characteristics in women with uRPL. Another subset of uNK cells, the IL-22-producing NK cells (NK22), has been found to be differently regulated in women with RPL. An increase in the proportion of NK22 cells has been detected in the endometrium, as well as in the peripheral blood, of women with unexplained RPL compared with women with unexplained infertility [136,137]. However, uNK cells in the decidua of women with unexplained RPL have a significantly lesser gene and protein expression of IL-22 than normal women [138]. Further investigation is needed to fully clarify the role of endometrial IL-22 and NK22 in RPL. Conflicting data obtained in uNK determinations in relation to RPL still exist and this could be due to differences in the populations studied or in the assay methodologies used or the complexity of subtypes of uNK cells in the endometrium and decidua. This point is particularly relevant, since there is the need for a standardization in the measurement of uNK in women with RPL, in order to better characterize several still-uncovered aspects concerning the actual role of these relevant cells in RPL [93,139], with potentially useful applications also in clinical practice. In fact, at present it still uncertain what the prognostic value of the determination of uNK count and parameters in women with uRPL are [140]. 

There is still an open debate on whether, or to which extent, the peripheral NK cell subsets correlate with the endometrial NK cells in normal women, in infertile women, and in women with RPL, even though there is evidence that peripheral NK cells could reflect, at least to some degree, the local endometrial environment [131,141,142,143,144,145,146]. This issue is beyond the scope of the present article, which is focused on the local endometrial immune environment in RPL. 

The dysregulation of NK cells in the endometrium and decidua of women with RPL has potentially important consequences, leading to fatal pregnancy impairment. Particularly relevant in this context are the maintained cytotoxicity of uNK [23], the impaired capacity of uNK to properly interact with the specific HLA expressed by trophoblast [146,147,148], the impaired capacity of uNK to effectively participate in the full vascular remodeling of the uterine spiral arteries [129,149], the impaired capacity of uNK in limiting T cell cytotoxicity [61], and a disturbed cytokine production pattern by uNK [143,150,151].

Therefore, evidence is accumulating that in uRPL, uNK are profoundly dysregulated in their major pregnancy-promoting functions.

#### 3.2.3. Uterine Dendritic Cells (uDCs)

The discovery of the important role of uDCs as powerful pregnancy-promoting cells at the maternal–fetal interface has prompted an increasing interest in unraveling the potential involvement of these immunoregulatory cells in RPL. There is evidence that abnormalities in uDCs are associated with early pregnancy disorders. In a transgenic mouse model, depletion of uDCs impairs embryo implantation and results in embryo resorption [152]. This action could be exerted independently of the immunoregulatory effect of uDCs, suggesting that uDCs could exert other supporting roles in implantation in addition to their tolerogenic action [152]. In human studies, it has been shown: (1) that elevated levels of Th1-inducing myeloid DCs and reduced levels of tolerogenic CD200+ DC subsets in peripheral blood are associated with RPL [153]; (2) that mature (non-tolerogenic) dendritic cells in gestational decidua and placenta are increased and immature tolerogenic uDCs are decreased in women with RPL compared with normal women [154,155]; (3) that in women with RPL there is a downregulation of ILT4+ tolerogenic DCs in both peripheral blood and endometrium [156]. 

#### 3.2.4. Tregs

Considerable information is available on the potential role of Treg alterations in RPL. As in the case of uNK, the emerging role of these cells in embryo implantation and in maternal immunotolerance in pregnancy has prompted a growing research interest in this issue. Studies carried out on animal models revealed that depletion of CD4+CD25+ Tregs resulted in the greatest increase in miscarriage rates [157,158], which were linked to the expansion of activated CD4+ and CD8+ T cells occurring only in the uterine draining lymph nodes [158]. Conversely, the transfer of Tregs could prevent miscarriage in abortion-prone mice [159,160]. Interestingly, this action is of particular relevance at the time of implantation and in early pregnancy with a less marked effect in more advanced gestation [20,108,161], suggesting that Tregs play a key role mainly at implantation and in the very early stages of pregnancy. Studies of Tregs in women with RPL are in substantial agreement with the findings obtained in animal models. Women with RPL have reduced Tregs in peripheral blood [162,163,164], as well as in their endometrium and decidua, compared with normal women [163,165,166,167]. The reduction in Tregs in women with uRPL is paralleled by an increase in Th17 cells [166,168], suggesting the presence of an imbalance in Tregs/Th17 cells in uRPL [169,170,171]. The likely involvement of Tregs in uRPL is also supported by the studies carried out on the forkhead box P3 (FOXP3), a transcription factor essential for the development and function of Tregs. There is an increased occurrence of the following mutant genotypes and alleles of FOXP3 single nucleotide polymorphisms in women with uRPL compared with control women: rs2232365, rs3761548, rs5902434, and rs2294021 [172,173]. Moreover, FOXP3 expression was reduced in peripheral blood and decidua in women with uRPL compared with normal women [163]. In women with both uRPL and sporadic miscarriages, abnormalities in the normal function exerted by Tregs in uncomplicated pregnancies have been identified including: (1) higher numbers of Tregs from women with uRPL than normally fertile women were required to inhibit proliferation of CD4^+^CD25^-^ effector cells in response to paternal allostimulation [56], and (2) the Helios+ Tregs—a supposed functionally active subset [174]—are reduced in the decidua of women with sporadic miscarriage with normal embryo karyotype [165]. This last finding suggests that also some (or several) cases of sporadic miscarriage, with normal fetal chromosome content, might be associated with the immune etiology of miscarriage [165]. Since Tregs are involved in the downregulation of an excessive inflammatory response toward the implanting embryo and the fetus, it is conceivable that dysregulation and/or dysfunction of these cells could be a relevant factor leading to RPL. Finally, it cannot be excluded that unstable dysregulated Tregs could directly be involved in fetal rejection, since Tregs are highly plastic, allowing them to transdifferentiate, under specific conditions, into effector T cells [175]. 

### 3.3. Endometrial Cytokine Imbalance in RPL

Consistent experimental evidence indicates that RPL is associated with an imbalance in the endometrial and decidual cytokine environment typical of the normal pregnancy. The general concept is that in women with uRPL, as well in animals prone to RPL, a dysregulated production of several cytokines relevant for implantation and early pregnancy promotion occurs in both immune and non-immune cells present or recruited into the endometrium and decidua. This generates an unfavorable cytokine environment which, in turn, can impair the tolerance of the maternal immune system towards the trophoblast and lead to the rejection of the conceptus. Owing to the complexity of the endometrial cytokine network, which additionally undergoes profound changes in pregnancy, it is difficult to identify all the cytokines involved in the determination of uRPL and define the relative importance of each specific substance. Moreover, it can be difficult to exactly determine the specific cell source of these factors, which are probably secreted by a wide variety of both immune- and non-immune cells. Despite the above limitations, several selected cytokines are believed to play a significant role in this context. These cytokines include at least IL-1, IL-1α, IL-1β, IL-2, IL-4, IL-6, IL-7, IL-8, IL-10, IL-12, IL-17, IL-18, IL-22, IL-23, IL-27, TGF-β, TGF-β1, IFN-γ, TNF-α, LIF, and MIF. The reported changes of these cytokines in the endometrium and/or decidua in women with RPL compared with normal women are summarized in Table 5. 

Even though the results are sometimes conflicting, due to methodological differences in cell/tissue preparation or separation, as well as in assay techniques, the emerging picture clearly defines a potentially relevant role for selected molecules, such as IL-1α and β, TGF-β, TNF-α, IL-10, and LIF. However, the potential relevant contribution of many other cytokines in RPL cannot be excluded. 

Since the results concerning the variations in expression/production of many cytokines by the endometrium and decidua in RPL are still conflicting, possibly owing to the above mentioned limitations, some more details will be given for few selected substances for which enough agreement exists. 

It is believed that uRPL is associated with a decrease in the decidual TGFβ. This is due to a reduced production of TGFβ by decidual dendritic cells [176], to a reduced mRNA expression of TGFβ [166], and to a reduced proportion of TGFβ^+^ Tregs in the decidua [177] of women with RPL compared to normal control women. Since TGFβ has a recognized pregnancy-promoting role in implantation and early pregnancy development, it is likely that a defective production of this cytokine can be involved in uRPL of immune etiology. 

An increase in endometrial levels and decidual mRNA and protein expression of IFN-γ has been consistently found in women with RPL [177,178,179,180,181]. RPL in presence of an excess of IFN-γ could be the consequence of an excessive inflammatory reaction in the decidua, triggered by IFN-γ; moreover, IFN-γ has a toxic effect on the embryo by inducing apoptosis [33] and, therefore, RPL.

IL-10 contributes, together with other cytokines and immunoregulatory substances such as TGFβ and IDO, to the differentiation of DC into the tolerogenic phenotype, which, in turn, regulates the expansion of Treg population in the decidua. The observed decrease in IL-10 expression and production in the decidua of women with uRPL [176,177,178,179,180] can hamper this immunoprotective action.

The results obtained by studying the local uterine abnormalities in cytokine levels/production are only in partial agreement with those found in studies carried out on peripheral blood and cells, leaving open the question on whether it could be possible to obtain markers suggestive of immunologic RPL from the peripheral blood [136,143,151,182,183,184]. Additionally, conflicting results were found in several meta-analyses aimed at evaluating the importance of specific SNP for selected cytokines in women with RPL [185,186]. Therefore, the immunogenetic contribution of cytokine gene variations in RPL needs further evaluation to be clearly determined [187].

## 4. Conclusions and Future Directions

In the last three decades, enormous progress has been made in the comprehension of the mechanisms underlying the maternal acceptance of the genetically and immunologically different embryo. The emerging picture can be synthesized as follows: (a) it has become clear that the maternal immune system is intimately involved in the establishment, maintenance, development, and termination of the normal pregnancy. In this context, it is logical and highly plausible that the immune system has been used to regulate all the phases of the pregnancy and, possibly, of reproduction in evolved animals; (b) multiple mechanisms have been developed to prevent the maternal immune dysfunction leading to the loss of pregnancy. These mechanisms are aimed to induce an overall immunological tolerance of the mother towards the fetus while maintaining full immunological reactivity against all the other foreign antigens. To reach this objective, the maternal immune system needs to undergo a modulation which involves many, if not all, the major cellular and molecular components of the adaptive and innate immune systems, assigning to each of them specific, although sometimes redundant, duties; (c) the involvement of the maternal immune system is not limited to a correct immunologic dialogue at the maternal–fetal interface, but is extended to the uterine (endometrial) tissue breakdown, vascular remodeling, and placentation; (d) the trophoblast actively participates in its own survival and modulation of the maternal immune response to its presence; in this respect, its behavior is more similar to that of a tumor rather than to a transplant; (e) the maternal immune response to the conceptus undergoes considerable changes according to the gestational age. In fact, recent evidence supports the concept that the implantation of the embryo evokes an initial, early, inflammatory reaction which is promptly followed by the establishment of an anti-inflammatory decidual environment, allowing the survival of the conceptus and the progression of the pregnancy. A final, even more powerful, inflammatory reaction occurs in late gestation and results in labor and the delivery of the fetus. All of these events fully involve the maternal immune system.

In this context, it is very likely that derangements of the immune system can be operative in at least some women with unexplained RPL, in which no other clinical causes or explanation can be found. In these women, it is highly likely that an immune dysfunction occurs in the endometrium and in the decidua, which represents the major maternal–fetal interface and in which significant immunologic changes are known to physiologically occur. However, it is possible that different abnormalities in the immune system lead to the same final effect—the RPL—in different women; in other words, it is possible that different immunological modes of RPL exist. Some of these modes are summarized in Table 6 and Figure 1c. 

Future research is needed to answer this relevant question and to help develop effective immunologic treatments for this subset of women with RPL. Considerable progress is needed in this area. In fact, the currently available treatments for RPL of supposed immune etiology are rather limited, empiric in the majority of cases, and with low efficacy, with few exceptions, such as aspirin/aspirin plus heparin when used in the case of RPL in women with antiphospholipid antibodies [194]. Other treatments based on a potentially reasonable immune background, as in the case of intravenous immunoglobulin (IVIg), have been shown to have a potential efficacy in selected groups of women with RPL and when initiated before pregnancy [195]. 

Future perspectives in the treatment of RPL of immune etiology could be aimed to correct abnormal decidualization as well as dysfunctions of the immune mechanisms occurring in the endometrium and decidua, on the basis of the emerging evidence. As an example, the effects of seminal plasma on the expansion of Tregs could allow an innovative therapeutic approach, that is already being used in assisted reproductive technologies (ART), even though with still controversial results [196,197,198].

Another emerging area of investigation and potentially useful treatment is represented by situations in which RPL is associated with chronic inflammation of the endometrium (chronic endometritis, CE). CE, that is still poorly considered in most diagnostic protocols for RPL, in a recent study has been found in a substantial proportion (27%) of women with unexplained RPL [199]. This finding confirms the results of previous studies in which CE has been detected in 13%–56% of women with RPL [200,201] and supports the concept that CE could be a not negligible cause of RPL. The suggested mechanism by which CE could determine RPL is an alteration of the normal decidualization of endometrial stromal cells [202]. Since proper treatment of CE in RPL women can lead to improvement in live birth rates [203], the clear detection of this condition can be beneficial at least in a subset of these patients.

## Figures and Tables

**Table 1:** Simplified summary of the major changes characterizing the decidualization of the endometrium.

**Morphological (Tissutal and Cell) Changes**
Secretory transformation of endometrial glands, compaction of surface epithelial cells, stromal edema, stromal cell proliferation, differentiation of fibroblast-like stromal cells into epithelioid-like cells, massive leukocyte infiltration (mainly uterine natural killer cells and mast cells), spiral arteries remodeling
**Extracellular Matrix Changes**
Increased production by decidualized stromal cells of collagen IV, fibronectin, laminin, decorin, heparan-sulphate proteoglycans, IGFBP-1, PRL, LEFTY-2, αvβ3 integrin, osteopontin
**Changes in the Expression of Genes Involved in the Following Cellular Functions**
Cell cycle regulation, cytoskeleton remodeling, oxidative stress response, ion and water transport, response to steroid hormone, deposition of extracellular matrix (ECM), modulation of transcription, epigenetic patterning, post-translation modification, growth factor, angiogenesis, cytokine and chemokine signaling, immune modulation
**Cell Signaling and Pathways Involved in the Decidualization**
cAMP/PKA pathway, progesterone signaling pathway, Nodal pathway, Notch signaling pathway, ERK1/2 pathway, WNT/β-catenin pathway, cSRC pathway, JAK-STAT pathway, lipid signaling (endocannabinoid system), TGFβ signaling pathway, BMP2-WNT4 signaling cascade, phosphatidylinositol 3-kinase/AKT pathway, Ras/Raf1/MAPK pathway, EPAC1 and EPAC2 signaling

LEFTY-2: left-right determination factor 2; IGFBP-1: insulin like growth factor binding protein-1; PRL: prolactin; cAMP: cyclic adenosine monophosphate; PKA: protein kinase A; ERK1/2: extracellular signal-regulated kinase 1/2; JAK: Janus kinase; STAT: signal transducer and activator of transcription; TGFβ: transforming growth factor-β; BMP-2: bone morphogenetic protein-2; AKT: protein kinase B; MAPK: mitogen-activated protein kinase; EPAC1 and 2: exchange protein directly activated by cAMP 1 and 2.

**Table 2:** Overview of the relevant cells of the innate immune system in the endometrium and decidua with their major reproductive functions.

Cell Type	Phenotype	Density (%) of Leukocyte Population in the Endometrium	Changes during the Menstrual Cycle and in Early Pregnancy	Preferential Location in the Endometrium	Relevant Molecules Secreted	Suggested Functions	References
Uterine Natural killer Cells (uNK)	CD3^−^CD^56bright^CD16^−^ (predominant phenotype)	30%–40% of stromal cells 70% of endometrial leukocytes in the late luteal phase (LP) and in early pregnancy	Progressively increase from the follicular phase (FP) to the LP. Maximal density in late LP and in gestational decidua	Surround the arteries and the glands	IFN-γ, VEGF, PlGF, TGF-β, TNF-α, IL-10, GM-CSF, IL-1β, LIF, CSF-1, AP-2	Tissue (spiral arteries) remodeling, enhancement of angiogenesis, control of trophoblast invasion	[10,20,24,25,26,27,28,29]
Macrophages (Mφ)	CD68^+^	20%–25% of total leukocytes in the decidua	Progressively increase from the FP to the LP. Maximal density before menstruation and in pregnancy. Acquire tolerogenic phenotype	Scattered throughout the endometrium; preferentially found around the glands and at implantation site	TGF-β, IL-10, IDO, PGE_2_	Involved in corpus luteum maintenance, blastocyst implantation, spiral arteries remodeling, control of trophoblast invasion, protection of the fetus against intrauterine infection	[20,29,30,31,32,33,34,35]
Mast Cells (MCs)	MC_T_; MC_TC_; MC_C_ (endometrial MCs)	3%–5% of total endometrial cells;	Unchanged throughout menstrual cycle; changes in phenotype during the menstrual cycle; activated in the early and midluteal phase	More prominent in the basal endometrial compartment	VEGF	Initiation of menstruation Enhance tissue and spiral artery remodeling, support implantation and angiogenesis	[20,36,37]
Uterine Dendritic Cells (DCs)	CD1a+ (immature, tolerogenic DCs); CD83+ (mature DCs)	Density of immature DCs in the endometrium is higher than that of mature DCs; DC 1%–2% of the immune cells in the decidua	Immature DCs increase from FP to LP; peak in the menstrual phase (controversial finding); No changes in mature DCs with the menstrual cycle	Both mature and immature DCs are found mainly in the basal layer of the endometrium in the LP; Scattered through the gestational decidua In mice grouped in cluster-like structures	TGF-β, IL-10, IDO	Involved in the maternal acceptance of the embryo, trophoblast invasion and differentiation; Uterine remodeling, angiogenesis; Determine the differentiation of T cell progenitors into Tregs and expansion and activation of Tregs	[29,33,38,39,40,41,42,43]
Neutrophil (N) Granulocytes	CD11b+; CD16b+; CD66c+	1% to 6%–15% of endometrial cells	Considerably increase in the late (premenstrual) LP	Endometrial Stroma	CCL2, CXCL8, TNF-α, IL-6, VEGF	Menstruation, tissue breakdown and repair, Proangiogenic and tolerogenic in the pregnant decidua	[44,45,46,47]

**Table 3:** Overview of the cells of the adaptive immune system in the endometrium and decidua with their major reproductive functions.

Cell Type	Phenotype	Density (%) of Leukocyte (CD45+) Population in the Endometrium	Changes during the Menstrual Cycle and in Early Pregnancy	Preferential Location in the Endometrium	Relevant Molecules Secreted	Suggested Functions	References
**BLymphocytes**	CD45+ CD19+	0.2%–4.5%	Very slight increase in late LP	Clusters among stromal cells in the perimenstrual period	IL-10	Still undetermined; Potentially implicated in early pregnancy	[20,47,48]
**T Lymphocytes**	CD45+ CD3+	1%–2% to 28%	Reportedly decreased or unchanged from follicular phase (FP) to luteal phase (LP)	Lymphoid aggregates; scattered throughout epithelium and stroma	Variable according to the specific cell subset	Protective or harmful for the embryo according to the specific cell subset	[24,29,48,49]
**T Helper Lymphocytes** **(Th1, Th2, Th17, Tregs)**	CD45+ CD3+ CD4+	3.8%–21.4%	No clear variations reported	Present in uterine mucosa as unique aggregates surrounding a B cell core	TNF-α and IFN-γ (by Th1); IL-4 (by Th2); IL-8 (by Th17)	Th1 produce inflammatory cytokines; Th2 produce anti-inflammatory cytokines; Th17 has pro-inflammatory effects (for Tregs see the dedicated section in the table)	[33,48,50]
**T Cytotoxic Lymphocytes**	CD45+ CD3+ CD8+	4.4%–34.5% to 66%	Significantly decrease from FP to LP	Lymphoid aggregates	Release cytotoxic substances (granzymes, perforin)	Potentially harmful to the embryo. Blocked in successful pregnancy	[47,48,49,51]
**Tregs**	CD4+ CD25+ FOXP3+	Not clearly defined in humans	Expansion in preimplantation endometrium; Increase in the decidua at implantation site and in early pregnancy until midgestation	Insufficient data in human preimplantation endometrium	Galectin-1, TGF-β, IL-10, HO-1	Essential in the control of an excessive maternal inflammatory response at the implantation site; Involved in maternal immune tolerance to fetal allograft particularly in early pregnancy; Block maternal effector T cells Involved in the regulation of maternal vascular remodeling	[20,33,49,50,52,53,54,55,56,57]

CD: cluster of differentiation; FP: follicular phase; LP: luteal phase; IFN-γ: interferon gamma; VEGF: vascular endothelial growth factor; PlGF: placental growth factor; TNF-α: tumor necrosis factor alpha; IL-10: interleukin-10; GM-CSF: granulocyte macrophage colony stimulating factor; IL-1β: interleukin-1β; LIF: leukemia inhibitory factor; CSF-1: colony-stimulating factor 1; AP-2: endocytic adaptor protein 2; IDO: indoleamine 2,3-dioxygenase; PGE2: prostaglandin E2; CCL2: chemokine C-C motif ligand 2; CXCL8: C-X-C motif ligand 8; IL-6: interleukin-6; IL-4: interleukin-4; IL-8: interleukin-8; HO-1: heme oxygenase-1.

**Table 4:** Proposed major mechanisms underlying maternal immune tolerance towards the embryo in normal pregnancy in the endometrium and decidua.

Mechanism	Effect	References
Increased secretion of LIF and IL-1β by uterine macrophages	Enhancement of embryo attachment to endometrial epithelium	[58]
TGFβ production by maternal decidual macrophages	Suppression of EVT rejection mediated by NK cells	[59]
Production by macrophages of TGFβ, IL-10, IDO, and PGE2	Immunoinhibitory and pro-tolerance actions	[33]
Specific characteristics of uNK cells in the endometrium and of dNK in pregnancy	Acquisition of a regulatory role rather than classic cell killing ability	[21,27,60]
dNK cells dampen Th17 cells through the production of IFN	Suppress Th17-induced inflammatory response	[61]
Galectin-1 production by dNK cells	Induces apoptosis of activated CD8+ T cells	[62,63,64]
Endometrial recruitment of innate immune cells (Mφ, DCs, and granulocytes) triggered by seminal fluid before implantation	Activation and expansion of Tregs which, in turn, creates a uterine microenvironment favorable for embryo implantation and enhances maternal tolerance towards paternal MHC antigens	[21,54,65,66,67]
Induction and expansion of tolerogenic DCs phenotype	Involved in the control and activation of Tregs	[33,54,68,69]
Reduced production of IL-12 by DCs	Priming of decidual CD4+ cells into a Th2 phenotype	[70,71]
EVT cells express HLA-C, HLA-E, and HLA-G but not HLA-A and HLA-B Lack of expression of MHC molecules by syncytiotrophoblasts	Inhibition of cytolytic activity of dNK cells against the trophoblasts Induction of NK senscence-Enhanced apoptosis of activated CD8+ cells	[70,72,73,74,75]
Trophoblast production of exosomes	Downregulation of maternal immunity towards the trophoblast	[76]
Development of a specific cytokine and chemokine network in the endometrium and decidua	Achievement of a correct immune cell recruitment and dialogue favoring embryo implantation and proper pregnancy evolution	[77]
Presence of asymmetric maternal Abs against paternal antigens	Bind trophoblast but are unable to trigger destructive immune response	[78]
Increased production of PIBF	Upregulation of Th2 cytokines production. Downregulation of dNK activity. Increase the production of Glycodelin A which induces apoptosis in T cells. These effects can contribute to trophoblast immune protection	[66,79]
Increased progesterone production in LP and in pregnancy	Expansion of Tregs and enhancement of their immunosuppressive actions	[33,78]
hCG production by syncytiotrophoblast	Recruitment of Tregs at the maternal–fetal interface	[80]

EVT: extravillous trophoblast; HLA: human leukocyte antigen; MHC: major histocompatibility complex; Mφ: macrophages; DC: dendritic cells; PIBF: progesterone induced blocking factor; LP: luteal phase; hCG: human chorionic gonadotropin; Abs: asymmetric antibodies.

**Table 5:** Summary of the major changes observed in cytokine expression/levels in the endometrium and decidua in women with RPL compared with normal women.

Cytokine	Tissue/Cells	Type of Variation	Expression/Production	Methods of Detection	References
IL-1 (α and β)	Decidua	Decrease	IL-1 pathway gene expression	Microarray	[188]
IL-1α	Endometrium	Decrease	mRNA expression	RT-PCR	[189]
IL-1β	Endometrium Endometrium	Increase Decrease	Tissue secretion mRNA expression	ELISA RNase protection assay	[190] [191]
IL-2	Decidua	Increase	Cytokine production	ELISA	[176]
IL-4	Decidua Decidual T cells	Decrease Decrease	mRNA and protein expression Cytokine production	ELISA + RT-PCR ELISA	[178] [179]
IL-6	Decidua Endometrium Endometrium	Increase Decrease Decrease	mRNA and protein expression mRNA expression mRNA expression	RT-PCR RT-PCR RNase protection assay	[166] [189] [191]
IL-7	Decidual stromal cells	Increase	Cytokine expression	IHC	[170]
IL-8	Decidua	Increase	IL-8 pathway gene expression	Microarray	[188]
IL-10	Decidua Decidua Decidua Decidual Tregs Decidual T cells	Decrease Decrease Decrease Decrease Decrease	mRNA and protein expression Cytokine production Protein expression Cytokine expression Cytokine production	ELISA + RT-PCR ELISA RT-PCT Flow cytometry ELISA	[178] [176] [180] [177] [179]
IL-12	Endometrium	Increase	Tissue levels	ELISA	[181]
IL-17	Decidua Decidua	Unchanged Increase	mRNA and protein expression mRNA and protein expression	qRT-PCR + IHC +WB RT-PCR + WB	[138] [167]
IL-18	Endometrium Endometrium	Increase Increase	Tissue levels Tissue secretion	ELISA ELISA	[181] [190]
IL-22	Decidua	Decrease	mRNA and protein expression	qRT-PCR + IHC +WB	[138]
IL-23	Decidua Decidua	Unchanged Increase	mRNA and protein expression mRNA and protein expression	qRT-PCR + IHC +WB RT-PCR + WB	[138] [167]
IL-27	Decidua	Decrease	mRNA and protein expression	qRT-PCR + WB	[192]
TGF-β	Decidual Tregs	Decrease	Cytokine expression	Flow cytometry	[177]
TGF-β1	Decidua Decidua	Decrease Decrease	mRNA and protein expression Cytokine production	RT-PCR + ELISA ELISA	[166] [176]
IFN-γ	Endometrium Decidua Decidua	Increase Increase Increase	Tissue levels Cytokine production mRNA and protein expression	ELISA ELISA RT-PCR + ELISA	[181] [176] [178]
TNF-α	Decidua	Increase	mRNA and protein expression	RT-PCR + ELISA	[166]
LIF	Endometrium Endometrium Decidual T cells	Decrease Increase Decrease	Tissue levels mRNA expression Cytokine production	ELISA RT-PCR ELISA	[181] [193] [179]
MIF	Endometrium Decidual T cells	Decrease Decrease	Tissue levels Cytokine production	ELISA ELISA	[181] [179]

IL-2: interleukin-2; IL-4: interleukin-4; IL-6: interleukin-6; IL-7: interleukin-7; IL-8: interleukin-8; IL-12: interleukin-12; IL-17: interleukin-17; IL-18: interleukin-18; IL-22: interleukin-22; IL-23: interleukin-23; IL-27: interleukin-27; MIF: macrophage migration inhibitory factor.

**Table 6:** Summary of the suggested major immunologic alterations in the endometrium and potential pathogenetic mechanisms leading to RPL.

Suggested Alteration	Potential Pathogenetic Mechanism(s)
Abnormal decidualization of the ESC	Altered control of the local trafficking of immune cells by ESC; Altered modulation of the inflammatory response to the implanting embryo; Dysregulation in cytokine production
Abnormal killer immunoglobulin receptor (KIR)–uNK interaction	Abolished or reduced prevention of the maternal immune rejection of the conceptus
Increased toxicity of uNK	Impaired capacity to limit T cell cytotoxicity; Potential toxicity toward the cytotrophoblast; Abnormal cytokine production; Defective remodeling of the spiral arteries
Abnormal number/function of endometrial/decidual Tregs	Lack of inhibition of Th1 and Th17 cells; Defective/absent resolution of the inflammatory reaction consequent to initial embryo implantation; Reduction or abolition of the maternal immunologic tolerance toward the fetus; Defective remodeling of the spiral arteries
Limited differentiation of macrophages toward the M2 immunophenotype	Reduced production of tolerogenic cytokines (IL-10, TGF-β)
Lack of immature dendritic cells	Lack of the expansion of Treg population
Activation of lymphocytes T effector	Immune attack to the trophoblast
Aberrant local cytokine network	Development of a local immunomodulatory environment unfavorable to maternal tolerance toward the conceptus
